# More hip complications after total hip arthroplasty than after hemi­arthroplasty as hip fracture treatment: analysis of 5,815 matched pairs in the Swedish Hip Arthroplasty Register

**DOI:** 10.1080/17453674.2019.1690339

**Published:** 2019-11-18

**Authors:** Susanne Hansson, Erik Bülow, Anne Garland, Johan Kärrholm, Cecilia Rogmark

**Affiliations:** a Department of Orthopaedics, Lund University, Skåne University Hospital, Malmö;; b The Swedish Hip Arthroplasty Register, Registercentrum Västra Götaland, Gothenburg;; c Department of Orthopaedics, Visby Hospital, Visby;; d Department of Orthopaedics, Institute of Surgical Sciences, Uppsala University, Uppsala;; e Department of Orthopaedics, Institute of Clinical Sciences, Sahlgrenska Academy, University of Gothenburg, Sweden

## Abstract

Background and purpose — Total hip arthroplasty (THA) is increasing as treatment of displaced femoral neck fractures. Several studies compare hemiarthroplasty (HA) with THA, but results vary and few studies report on medical complications. We examined the outcome of THA and HA with a focus on medical complications, hip complications, and death.

Patients and methods — Data from the Swedish Hip Arthroplasty Register on 30,953 acute hip fracture patients treated with cemented THA or HA in 2005–2011 were cross-matched with Statistics Sweden for socioeconomic data and with the National Patient Register for diagnostic codes representing medical complications within 180 days or hip complications within the study period. Propensity score matching was used to create comparable groups based on age, sex, income, level of education, marital status, Elixhauser index, and year of surgery. Logistic regression models were created for each outcome.

Results — 81% were treated with HA, 73% and 71% were female (HA and THA respectively). Matching resulted in 2 groups of 5,815 patients each. THA was associated with fewer medical complications (OR = 0.83; 95% CI 0.76–0.91) and lower 1-year mortality (OR = 0.42; CI 0.38–0.48), but more hip complications (OR = 1.31; CI 1.20–1.43).

Interpretation — THA as treatment of hip fracture was associated with more hip-related complications than HA. The results on mortality and medical complications are, rather, influenced by residual confounding than by the implant design per se. An expansive use of THAs for hip fracture treatment, at the expense of HAs, is not recommended based on our findings if hip complications are to be avoided.

Hemiarthroplasty (HA) or internal fixation have been the main alternatives for treatment of displaced femoral neck fracture. Total hip arthroplasty (THA) has increased in popularity as fracture treatment (Kärr­holm et al. 2018). Several studies have compared HA with THA, but the results vary. Age, activity level, health, and supposed remaining lifespan of the patient are factors influencing the choice between THA and HA in clinical practice.

HAs may have a lower risk of dislocation (Burgers et al. 2012, Rogmark and Leonardsson 2016). However, since the head articulates directly against the cartilage, patients receiving HA may develope acetabular erosion (Avery et al. 2011, Wang et al. 2015). THA often entails longer surgeries (Blomfeldt et al. 2007, van den Bekerom et al. 2010). In contrast, some studies have shown THA to be associated with lower mortality (Avery et al. 2011, Hansson et al. 2017, Wang and Bhattacharyya 2017).

An adverse event is defined as an unintended injury or complication resulting in temporary or permanent disability, death, or prolonged hospital stay, and is caused by the healthcare management rather than by the natural disease process (Brennan et al. 1991, Merten et al. 2015). Adverse events implicate both medical and hip complications as well as death. Studies comparing THA and HA traditionally have focused on hip function and hip complications, but fewer report on medical complications. Earlier we found function after hip fracture to be affected not only by hip complications, but also by medical complications (Hansson et al. 2015), stressing the need to include both in the comparisons between implants.

We examined the difference in outcome between THA and HA with a focus on adverse events to provide support for the decision on which type of arthroplasty to use as treatment for femoral neck fractures. The outcomes studied were medical complications, hip complications, and death.

## Patients and methods

We performed an observational cohort study by cross-matching data from 3 national Swedish registers: the Swedish Hip Arthroplasty Register (SHAR) (Kärrholm et al. 2018), the Swedish National Patient Register (NPR) (Ludvigsson et al. 2011), and Statistics Sweden (Statistics Sweden 2018). Each included individual was identified in all the registers through the unique personal identity number given to all Swedish residents.

SHAR aims to register all hip arthroplasties in Sweden, covering all hospitals, both public and private, with a completeness of approximately 97% for emergency procedures. All types of reoperations are recorded continuously, revisions as well as any other open procedures. Closed reductions of dislocations are not recorded in the register. In 2012, the completeness of revision surgery reported to SHAR was 94% (Kärr­holm et al. 2018). Hemiarthroplasties have been recorded in the register since 2005.

Statistics Sweden is responsible for producing official statistics for Sweden, for example on income, education, and marital status of Swedish residents. NPR covers all hospital-based health care in Sweden from both private and public caregivers, including inpatient care, outpatient visits, and psychiatric care. Main diagnosis, secondary diagnosis, and external cause of injury are recorded as ICD-10 codes. The completeness of main diagnosis is almost 99% (Ludvigsson et al. 2011). Procedures are recorded as NOMESCO codes (NOMESCO 2011). An Elixhauser comorbidity index (Elixhauser et al. 1998) was generated from the ICD-10 codes in NPR.

**Figure 1.**
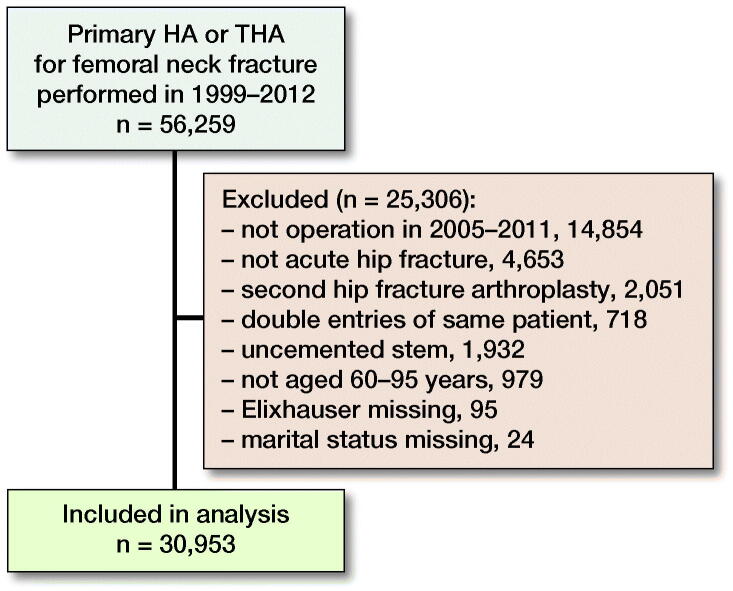
Flowchart of included and excluded patients.

A dataset was created with information from all 3 registers including patients with acute hip fracture treated with THA or HA in 2005–2012. To allow for at least 1 year of follow-up, patients with operations in 2012 were excluded. To avoid including the same patient more than once, only the first surgery was included for patients having 2 hip fractures treated with arthroplasty within the study period. Uncemented arthroplasties were uncommon (6%) and were excluded. Due to small numbers and difficulty in matching, patients aged less than 60 years or more than 95 years were excluded (Figure 1).

The presence of a large selection of ICD-10 codes and NOMESCO codes representing medical complications within 180 days after the hip fracture surgery or hip complications within the study period were noted and interpreted as the patient suffering a complication related to the hip fracture surgery (Appendix 1, see Supplementary data). In the NPR there is no information concerning laterality, which means that hip-related complications on either side were included. An adverse event was defined as the event of any medical and/or hip complication and/or death within 180 days post-surgery. Death within 1 year was noted as a separate outcome. From Statistics Sweden, information on income, education, and marital status was gathered as these may be potential confounders.

Pre-fracture characteristics were age, sex, income, education, marital status, and Elixhauser index. Because of its skewed distribution, income was transformed into a binary logarithm. The variable “age deviation” (age minus mean age) was created, allowing a more natural interpretation of the models. Elixhauser index was stratified into 4 groups for simplification (0, 1, 2, and 3+).

### Statistics

Patients treated with HA are generally older and have more comorbidities (Kärrholm et al. 2018), which we also saw in our material (Table 1). To be able to compare the 2 groups of patients, propensity score matching was used based on all the covariates (age, sex, income, education, marital status, Elixhauser index, and year of surgery). Exact matching would have been preferred if possible but was not an option due to the curse of dimensionality. Propensity score matching was our second choice. It allowed us to improve covariate balance between the samples. There was an imbalanced sample before matching (Table 1), but propensity score matching reduced this imbalance (Table 2).

**Table 1.** Patient demographics before matching. Values are frequency (%) unless otherwise specified

Factor	HA	THA	p-value
Sample size^a^	25,138 (81)	5,815 (19)	
Age, mean (SD)	84 (6.3)	75 (7.2)	< 0.001
Age groups			
60–69	644 (3)	1,342 (23)	
70–74	1,470 (6)	1,330 (23)	
75–79	3,515 (14)	1,464 (25)	
80–84	7,011 (28)	1,055 (18)	
85–89	12,498 (50)	624 (11)	
Women	17,829 (71)	4,215 (73)	0.02
Men	7,309 (29)	1,601 (28)	
Elixhauser index, mean (SD)	1.4 (1.4)	1.1 (1.3)	< 0.001
Income **^b^**, mean (SD)	16.88 (0.65)	16.94 (0.71)	< 0.001
Education			< 0.001
Primary school	15,327 (61)	2,998 (52)	
High school	6,795 (27)	1,908 (33)	
University	3,016 (12)	909 (16)	
Marital status,			< 0.001
Married	7,468 (30)	2,553 (44)	
Unmarried	2,187 (9)	612 (11)	
Divorced	2,885 (12)	938 (16)	
Widow/widower	12,598 (50)	1,712 (30)	
Year of surgery)			0.08
2005	3,033 (12)	721 (12)	
2006	3,384 (14)	714 (12)	
2007	3,534 (14)	817 (14)	
2008	3,765 (15)	862 (15)	
2009	3,824 (15)	853 (15)	
2010	3,775 (15)	893 (15)	
2011	3,823 (15)	955 (16)	

a % of total (n = 30,953)

b log_2_

**Table 2.** Patient demographics after propensity score matching. Values are frequency (%) unless otherwise specified

Factor	HA	THA	p-value
Sample size **^a^**	5,815 (50)	5,815 (50)	
Age, mean (SD)	77 (6.2)	75 (7.2)	< 0.001
Age groups			
60–69	644 (11)	1,342 (23)	
70–74	1,390 (24)	1,330 (23)	
75–79	2,011 (35)	1,464 (25)	
80–84	1,102 (19)	1,055 (18)	
85–89	668 (11)	624 (11)	
Women	4,157 (71)	4,215 (72)	0.2
Men	1,658 (29)	1,600 (28)	
Elixhauser index			0.04
0	2,206 (38)	2,355 (41)	
1	1,711 (29)	1,664 (29)	
2	1,083 (19)	1,023 (18)	
3+	815 (14)	773 (13)	
Income **^b^**, mean (SD)	16.89 (0.70)	16.94 (0.71)	0.002
Education			0.009
Primary school	3,126 (54)	2,998 (52)	
High school	1,886 (32)	1,908 (33)	
University	803 (14)	909 (16)	
Marital status			0.02
Married	2,476 (43)	2,553 (44)	
Unmarried	570 (10)	612 (11)	
Divorced	908 (16)	938 (16)	
Widow/widower	1,861 (32)	1,712 (29)	
Year of surgery			0.2
2005	712 (12)	721 (12)	
2006	818 (14)	714 (12)	
2007	813 (14)	817 (14)	
2008	850 (15)	862 (15)	
2009	828 (14)	853 (15)	
2010	874 (15)	893 (15)	
2011	920 (16)	955 (16)	
Age deviation, mean (SD)	0.77 (6.2)	–0.77 (7.2)	< 0.001

a % of total (n = 11,630)

b log_2_

Multivariable logistic regression models were created to compare THA with HA in terms of medical complications, hip complications, and 1-year mortality, with separate models for each outcome. The models included type of arthroplasty, the pre-fracture characteristics mentioned above, and year of surgery. The results are presented as odds ratios (OR), with 95% confidence intervals (CI).

### Ethics, funding, and potential conflicts of interest

The study was approved by the Regional Ethical Review Board in Gothenburg, Sweden (ref. 271-14). This work was supported by grants from the Southern Health Care Region, Sweden. No competing interests declared.

## Results

30,953 patients were included in the study. A majority were treated with HA (81%) and most of the patients were female, in both the THA and the HA group (73% and 71% respectively). On average, HA patients were 9 years older than THA patients. A higher percentage of HA patients were widowed whereas a higher proportion of THA patients were married. The THA and HA patients differed significantly (p < 0.001) on all variables except for sex and year of surgery (Table 1). After propensity score matching, 2 comparable groups of HA and THA patients were generated with 5,815 patients in each group (Table 2).

One-third of the hemiarthroplasties were of unipolar design, one-third were of bipolar design, and one-third were of unknown design. The surgical approach for a majority of the patients with both HA and THA was direct lateral (Hardinge or Gammer). The mean time to death, revision, or loss of follow-up was 2.5 and 3.5 years for HA and THA respectively (Table 3).

**Table 3.** Surgical data on THA and HA. Values are frequency (%) unless otherwise specified

Factor	HA	THA
Design of HA		
Unipolar	7,827 (31)	
Bipolar	7,507 (30)	
Unknown	9,804 (39)	
Surgical approach		
Hardinge	1,565 (6.2)	311 (5.3)
Moore	7,333 (29)	1,497 (26)
Gammer	7,000 (28)	1,890 (33)
Other/missing	9,240 (37)	2,117 (36)
Follow-up **^a^**, mean (SD)	2.5 (2.0)	3.5 (2.1)

**^a^** Time to death, revision or loss of follow-up

The most common medical complications were cardiovascular, pneumonia, and urinary tract infection. The most common specified hip complications were fracture surgery on femur, dislocation, and infection (Table 4). When we compared the matched populations of patients with THA with those treated with HA and adjusted for potential confounders, we found THA to be associated with fewer medical complications (OR = 0.83; CI 0.76–0.91) (Table 5, see Supplementary data) and more hip complications (OR = 1.31; CI 1.20–1.43) (Table 6, see Supplementary data). THA was also associated with a lower 1-year mortality than HA (OR = 0.42; CI 0.38–0.48) (Table 7, see Supplementary data). Income and education did not have a significant effect on outcome in any of the models, whereas status as widowed, divorced, or unmarried tended to be associated with a worse outcome, with some variations depending on the outcome studied.

**Table 4.** Frequency of medical and hip complications, n (%)

	THA	HA	All
**Medical complications**			
Cardiovascular	478 (8.2)	3,536 (14)	4,014 (13)
Pneumonia	124 (2.1)	1,065 (4.2)	1,189 (3.8)
Urinary tract infection	134 (2.3)	974 (3.9)	1,108 (3.6)
Cerebrovascular	79 (1.4)	578 (2.3)	657 (2.1)
Thromboembolic	131 (2.3)	372 (1.5)	503 (1.6)
Urinary retention	60 (1.0)	351 (1.4)	411 (1.3)
Renal failure	31 (0.5)	205 (0.8)	236 (0.8)
Stomach ulcer	41 (0.7)	183 (0.7)	224 (0.7)
Pressure ulcer	22 (0.4)	192 (0.8)	214 (0.7)
**Hip complications**			
Fracture surgery femur	311 (5.3)	1,283 (5.1)	1,594 (5.1)
Dislocation	316 (5.4)	776 (3.1)	1,092 (3.5)
Infection	232 (4.0)	859 (3.4)	1,091 (3.5)
Any reoperation	153 (2.6)	572 (2.3)	725 (2.3)
Prosthesis or implant extraction	189 (3.3)	494 (2.0)	683 (2.2)
Wound healing problems	116 (2.0)	402 (1.6)	518 (1.7)
Girdlestone, arthrodesis	8 (0.1)	56 (0.2)	64 (0.2)
Other hip complication	372 (6.4)	901 (3.6)	1 273 (4.1)
Other surgical complication	17 (0.3)	61 (0.2)	78 (0.3)

## Discussion

We found THA to be associated with more hip complications than HA in hip fracture patients. This undermines the basis for the last decade’s increase in the use of THA, namely that THA results in fewer reoperations (Hopley et al. 2010, Hansson et al. 2017, Xu et al. 2018). How could the controversy between both higher hip complication rate and lower reoperation rate for THA be explained? Whether to perform secondary surgery or not is often left to the discretion of the orthopedic surgeon. In the case of dislocations, pain, and acetabular erosion in HA cases, this implant can relatively easily be converted to a THA by adding an acetabular cup. The threshold to perform revision surgery with exchange of existing implant parts might be higher, due to concerns about bone quality and patient frailty. This, we speculate, might explain the controversy. The fact that 1 register study (Jameson et al. 2013) and several randomized controlled trials (RCTs) (Baker et al. 2006, Keating et al. 2006, van den Bekerom et al. 2010, Hedbeck et al. 2011) have not found any difference in revision/reoperation rate between THA and HA may also question the superiority of THA. 1 recent register study did actually find a higher revision rate after THA compared with HA, but the THA group consisted of more young patients, more uncemented stems, and posterior approaches—all risk factors for revision (Moerman et al. 2018).

Since THA is associated with longer surgery and more blood loss (Blomfeldt et al. 2007, van den Bekerom et al. 2010), the procedure could be presumed to be more strenuous on the patients and in turn lead to more medical complications. We found the opposite: THA was associated with fewer medical complications. Neither a large register-based study (Liodakis et al. 2016) nor smaller RCTs (Baker et al. 2006, van den Bekerom et al. 2010) comparing THA and HA have previously found a significant difference in rates of medical complications. We also found THA to be associated with lower mortality. This was also found in a register-based study of 70,000 patients (Wang and Bhattacharyya 2017). However, a recent meta-analysis of RCTs (Xu et al. 2018), found no difference in mortality comparing THA with HA. The conflicting results probably reflect that observational studies suffer from selection bias rather than that the chosen type of arthroplasty affects mortality. On the other hand, the RCTs are often underpowered to detect subtle differences in mortality. Large studies enable us to find statistically significant results due to the large sample size and subsequently narrow confidence intervals. Nevertheless, the clinical significance should guide our treatment decisions. Latent diseases, non-recorded abuse and depression, and in particular the lack of data on pre-fracture function and frailty, imply residual confounding. Consequently, our findings on mortality and medical complications have to be interpreted with caution.

As mentioned, THA was associated with more hip complications than HA. The meta-analysis by Xu et al. (2018) reported only on dislocation and infection. In that study, THA had a statistically significantly higher risk of dislocation but there was no difference in terms of infection. Previous RCTs have not been able to show a difference in hip complications between THA and HA (Baker et al. 2006, Hedbeck et al. 2011), probably due to smaller sample sizes. By including a large number of patients and a wide variety of diagnostic and procedural codes representing hip complications in the crosslinking with NPR, we reduced the risk of neglecting postoperative complications not reported to SHAR, for example closed reduction of dislocation. A caveat is that our lack of information on laterality means that a potential hip-related complication on the opposite side could be included, which, compared with previous studies, implies a slight overestimation of hip-related complications. These events could, however, be expected to be rare within the time frame studied and occur with about equal incidence regardless of use of a hemi- or total hip arthroplasty on the side of primary interest.

The higher risk of dislocation for THA could partly explain the higher risk of hip complications for THA in our study. It should be noted that, during the first years of our study, femoral heads with a diameter of 28 mm dominated in Sweden to gradually become replaced by 32 and 36 mm heads. The cross-matched dataset did not contain information on head size. Differences in implant selection might thus be another reason for variations in results between studies and may also be one explanation for changes over time.

To be able to compare patients treated with HA and THA, we used propensity score matching. After the matching procedure about one-third of the patients remained for analysis. The entire population of patients with THA and HA are only partly overlapping in terms of age and comorbidity. Therefore, the matching procedure aims to mimic comparison of the 2 methods in the middle group consisting of somewhat frail, sick, and functionally impaired elderly people. A majority of the patients in the fracture arthroplasty population are very frail with substantial impairment pre-fracture. Very few surgeons consider THA suitable for this group and including them would not be clinically relevant. At the other end of the spectrum are those who are healthy and active at the level of younger adults. For them hemiarthroplasty is not used, and again comparisons are not clinically relevant. Thereby we end up with a segment of assumingly comparable individuals, who are included in the ongoing clinical debate.

We chose to present the results as odds ratios rather than relative risks. The most prevalent outcome was medical complications, where the largest odds ratio was around 4 (Elixhauser 3+). To approximate the relative risk by this odds ratio would overestimate the true value by close to 80%. Almost all other odds ratios are close to 1, however. To interpret these as relative risks would therefore be possible, and not misleading.

The strength of our register-based study is the comparatively large sample of more than 11,000 patients matched with respect to demography, comorbidities, and socioeconomic factors. Since we included all patients between 60 and 95 years of age, irrespective of comorbidities and cognitive function, our results are applicable to almost the entire population of patients treated with hip fracture arthroplasty. RCTs on the other hand comprise much smaller samples and usually only include healthy, cognitively intact, and relatively active individuals, thus excluding most patients with hip fracture. A limitation of our study is the observational design and the inability to control for potential confounding factors not recorded in any of the registers used. An optimum comparison can only be done by randomization, where equal patient groups are studied, but with the limitations mentioned above. In addition, we do not account for patient-reported outcome, which is of paramount interest to fully understand the clinical outcome of a procedure.

Meta-analyses can be used to find statistically significant and clinically relevant differences between treatment options even though the separate studies are too small to show a difference on their own. Some of the results in the recent meta-analysis by Xu et al. (2018) have already been discussed. However, Xu’s study did not report on any medical complications and the only hip complications examined were revision, dislocation, and infection. Our study includes a large number of diagnostic codes representing hip-related complications as well as medical complications and therefore gives a more complete picture of the adverse events after hip fracture surgery.

Finally, one cannot assume that 1 single implant type will suit all patient groups. In terms of early mortality, a register study indicated that THA in femoral neck fracture cases was comparatively safe in healthy patients less than 80 years old in comparison with those who were older and had several comorbidities (Hailer et al. 2016). The functional benefits with THA suggested by some—but not all—randomized trials (Baker et al. 2006, Keating et al. 2006, Macaulay et al. 2008, Hedbeck et al. 2011) may lie within reach for such relatively “young old” and healthy individuals, given that proper rehabilitation is provided. For the biologically aged, and that is the largest group, HA stands out as a satisfactory alternative.

In conclusion, THA as treatment of hip fracture was associated with more hip-related complications than HA. This difference may partly be explained by the use of smaller heads in THA during the study period. Further studies including only contemporary implants are needed to elucidate this issue. In such studies patient-reported outcomes should preferably be included to enable studies of any trade-off between patient-reported outcome and hip-related complications. The results on mortality and medical complications are influenced by residual confounding, rather than by the implant design per se. We fail to see how THA, a more strenuous operation with more local complications, should be the explanatory factor for reduced mortality and morbidity. An expansive use of total hip arthroplasties for hip fracture treatment, at the expense of hemiarthroplasties, is not recommended based on our findings if hip complications are to be avoided.

### Supplementary data

The Appendix and Tables 5–7 are available as supplementary data in the online version of this article, http://dx.doi.org/10.1080/17453674.2019.1690339

## Supplementary Material

Supplemental Material
