# Supplementary material for: More hip complications after total hip arthroplasty than after hemi­arthroplasty as hip fracture treatment: analysis of 5,815 matched pairs in the Swedish Hip Arthroplasty Register
Source: Acta Orthop. 2019 Nov 18;91(2):133–8. doi: 10.1080/17453674.2019.1690339 (PMC7144190; doi:10.1080/17453674.2019.1690339)
Supplement: Supplemental Material [file IORT_A_1690339_SM3504.pdf]

Supplementary data

Appendix 1. Categorization of ICD-10 and NOMESCO codes representing medical and hip complications

Category of complication	ICD-10 and NOMESCO codes
**Medical complications**	
Cardiovascular	I11.x–I13.x, I20.x–I24.x, I25.3, I25.4, I25.6, I30.x–I33.x, I38.9, I39.8, I40.x–I41.x, I44.x–I50.x, I51.1–I51.4, I51.6–I51.9, I52.x, I70.2–I70.9, I71.0, I71.1, I71.3, I71.5, I71.8, I72.x, I73.9x, I77.0–I77.2, I77.6–I77.9, I79.x, I97.8, I97.9, I98.1, I98.8, I99.9, J81.9, T81.0, T81.1, T81.7
Pneumonia	J12.x–J22.x
Urinary tract infection	N30.0, N30.9, N39.0
Cerebrovascular	I60.x–I66.x, I67.6, I67.8, I67.9, I68.1–I68.8, I81.9, I82.0–I82.3
Thromboembolic	I26.x, I28.x, I80.x, I82.8, I82.9, I87.0
Urinary retention	R33.9
Renal failure	I12.0, I13.x, N17.x, N99.0
Stomach ulcer	K25.x, K26.x, K27.x, K29.8, K29.9
Pressure ulcer	L89.x
**Hip complications**	
Fracture surgery femur	M966F, NFJx
Infection	M00.0, M00.0F, M00.1, M00.2, M00.2F, M00.8, M00.8F, M00.9, M00.9F, M86.0F, M86.1F, M86.6, M86.6F, NFSx, T81.4, T84.5, T84.5F, T84.5X, T84.7, TNF05, TNF10
Dislocation	M24.3–4, M24.4F, NFH0–NFH30, NFH4x, NFH7–9, S73.0, T93.3
Any reoperation	NFA00–22, NFA31–32, NFCx, NFF01–12, NFF22, NFF32, NFF92, NFL09–19, NFL39–49, NFL69–99, NFM09–29, NFM49, NFM79–99, NFTx, NFWx
Girdlestone, arthrodesis	NFG09–49, NFG99, NFQ09
Extraction of prosthesis or implant	NFUx
Other hip complication	M24.5–6, M25.6, T84.0, T84.0F, T84.0X, T84.3, T84.4, T84.8, T88.8
Problems with wound healing	QDBx, QDE35, QDG30, T81.3
Other surgical complication	G57.0, G57.2–4, G57.8–9, G97.8–9, M96.8–9, S34.2, S74.x, T81.2, T81.5–6, T81.8, T81.8W, T81.9, T88.9

Table 5. Logistic regression model of medical complications

Factor	Odds ratio (95% CI)
Hemiarthroplasty	1
THA	0.83 (0.76–0.91)
Age deviation	1.04 (1.03–1.04)
Men	1
Women	0.66 (0.60–0.73)
Income (log_2)	0.98 (0.91–1.04)
Primary school	1
High school	1.09 (0.99–1.20)
University	1.14 (1.00–1.30)
Married	1
Unmarried	1.24 (1.07–1.44)
Divorced	1.11 (0.98–1.26)
Widowed	1.09 (0.97–1.22)
Elixhauser 0	1
Elixhauser 1	1.59 (1.42–1.77)
Elixhauser 2	2.44 (2.16–2.76)
Elixhauser 3+	4.15 (3.65–4.72)
Year of surgery	0.99 (0.97–1.02)

Table 6. Logistic regression model of hip complications

Factor	Odds ratio (95% CI)
Hemiarthroplasty	1
THA	1.31(1.20–1.43)
Age deviation	1.00 (0.99–1.00)
Men	1
Women	0.84 (0.76–0.93)
Income (log_2)	1.03 (0.96–1.10)
Primary school	1
High school	1.05 (0.95–1.16)
University	1.06 (0.93–1.21)
Married	1
Unmarried	1.10 (0.95–1.28)
Divorced	1.17 (1.03–1.32)
Widowed	1.09 (0.98–1.22)
Elixhauser 0	1
Elixhauser 1	1.10 (0.99–1.23)
Elixhauser 2	1.30 (1.15–1.47)
Elixhauser 3+	1.51 (1.32–1.73)
Year of surgery	0.90 (0.88–0.91)

Table 7. Logistic regression model of 1-year mortality

Factor	Odds ratio (95% CI)
Hemiarthroplasty	1
THA	0.42 (0.38–0.48)
Age deviation	1.04 (1.03–1.05)
Men	1
Women	0.56 (0.50–0.64)
Income (log_2)	1.03 (0.94–1.12)
Primary school	1
High school	1.02 (0.90–1.16)
University	0.83 (0.69–0.99)
Married	1
Unmarried	1.07 (0.88–1.30)
Divorced	1.20 (1.02–1.41)
Widowed	1.03 (0.89–1.18)
Elixhauser 0	1
Elixhauser 1	1.27 (1.09–1.48)
Elixhauser 2	2.13 (1.82–2.49)
Elixhauser 3+	3.85 (3.28–4.51)
Year of surgery	0.99 (0.96–1.02)
